# Efficient motif finding algorithms for large-alphabet inputs

**DOI:** 10.1186/1471-2105-11-S8-S1

**Published:** 2010-10-26

**Authors:** Pavel P Kuksa, Vladimir Pavlovic

**Affiliations:** 1 Department of Computer Science, Rutgers University, Piscataway, NJ 08854, USA

## Abstract

**Background:**

We consider the problem of identifying motifs, recurring or conserved patterns, in the biological sequence data sets. To solve this task, we present a new deterministic algorithm for finding patterns that are embedded as exact or inexact instances in all or most of the input strings.

**Results:**

The proposed algorithm (1) improves search efficiency compared to existing algorithms, and (2) scales well with the size of alphabet. On a synthetic planted DNA motif finding problem our algorithm is over 10× more efficient than MITRA, PMSPrune, and RISOTTO for long motifs. Improvements are orders of magnitude higher in the same setting with large alphabets. On benchmark TF-binding site problems (FNP, CRP, LexA) we observed reduction in running time of over 12×, with high detection accuracy. The algorithm was also successful in rapidly identifying protein motifs in Lipocalin, Zinc metallopeptidase, and supersecondary structure motifs for Cadherin and Immunoglobin families.

**Conclusions:**

Our algorithm reduces computational complexity of the current motif finding algorithms and demonstrate strong running time improvements over existing exact algorithms, especially in important and difficult cases of large-alphabet sequences.

## Background

**Figure 1:**
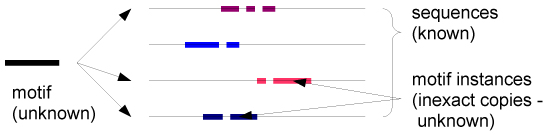
The motif search problem.

Finding motifs or repeated patterns in data is of wide scientific interest [1-4] with many applications in genomic and proteomic analysis. The motif search problem abstracts many important problems in analysis of sequence data, where motifs are, for instance, biologically important patterns. For example, elucidating motifs in DNA sequences is a critical first step in understanding biological processes as basic as the RNA transcription. There, the motifs can be used to identify promoters, the regions in DNA that facilitate the transcription. Finding motifs can be equally crucial for analyzing interactions between viruses and cells or identification of disease-linked patterns. Discovery of motifs in music sequences, text, or time series data is a fundamental, general means of summarizing, mining and understanding large volumes of data. For the purpose of this study, motifs are (short) patterns that occur in an exact or* approximate* form in all or most of the strings in a data set. Consider a set of input strings *S* of size *N* = |*S*| constructed from an alphabet Σ. The solution for the (*k*,* m,* Σ, *N*)-motif finding problem (Figure 1) is the set *M* of *k*-mers (substrings of length *k*), *M* ⊆ Σ*^k^*, such that each motif* a* ∈ *M*, |*a*| = *k*, is at minimum Hamming distance of at most *m* from all (or almost all) strings* s* ∈ *S*.

In this work, we focus on a deterministic, exhaustive approach to motif search. Exhaustive motif finding approaches are guaranteed to report all instances of motifs in a set of sequences, but are faced by the exponential complexity of such search. As a consequence, the problem quickly becomes intractable for even moderately long motifs and small alphabets. We present a new deterministic algorithm for finding common patterns with the search complexity that scales well with the size of the alphabet. Compared to existing algorithms in this class (e.g. [5,6]) that have strong dependency on the alphabet size and work with small-alphabet input, our algorithms significantly improve search efficiency in the important case of large-alphabet inputs (e.g. protein alphabet, extended DNA alphabet, etc.) and inputs of large length. As we show in the experiments, using both synthetic and real data, our algorithms are orders-of-magnitude faster than existing state-of-the-art deterministic search algorithms, especially on large-alphabet inputs (e.g., protein sequences). This result extends applicability of the exact motif search algorithms to more complex problems requiring analysis of biological sequence data modeled as strings over large alphabets. The problem of motif discovery has been tackled extensively over the past two decades [7]. Within the class of exhaustive methods, a number of approaches have been proposed, including graph methods (WINNOWER) [2], explicit trie traversal (MITRA) [5], explicit mapping (Voting algorithms) [8], suffix trees [6,9], sorting and enumeration [10], etc. Existing exhaustive algorithms use* explicit* exploration of the motif space and require time proportional to the size of the* neighborhood* of a *k*-mer, i.e. the number of *k*-mer sequences at Hamming distance of at most *m* from it. This size,  depends on the alphabet size, and can lead to high computational complexity and running times, as shown in Table 1.

**Table 1:** Exact algorithms for motif search

Algorithm	Time Complexity	Space Complexity
SPELLER [9]	*O *(*nN*^2^*V*(*k, m*))	*O *(*nN*^2^*/w*)
MITRA [5]	*O *(*knNV* (*k,m*))	*O *(*nNk*)
CENSUS [20]	*O *(*knNV* (*k,m*))	*O *(*nNk)*
Voting [8]	*O *(*nNV* (*k,m*))	*O *(*nV* (*m,k*))
RISOTTO [6]	*O *(*nN*^2^*V* (*k,m*))	*O *(*nN*^2^)
PMS [10]	*O *(*n*^2^*NV* (*k,m*))	*O *(*n*^2^*N*)

Explicit mapping (voting) algorithms proposed in [8] use an indicator array *V* of the maximum size |Σ|*^k^* to find motifs through* voting.* Each length-*k* substring observed in the input has at most one vote for each input sequence and gives this vote to all of its *V*(*k*, *m*) neighbors. The substrings that occur in every input string will receive *N* votes and will be included in the output motif set *M*. The algorithm takes *O*(*k^m^*^+1^|Σ|*^m^nN*) time and requires at least *O*(*k^m^*^+1^|Σ|*^m^nN*) space. The large space requirement of the algorithm restricts its usage to small values of *k* and *m*, as well as to small alphabet size |Σ|.

One of the most efficient exact algorithms for motif search, the mismatch tree (MITRA) algorithm [5], uses efficient trie traversal to find a set of motifs in the input strings. Under a trie-based computation framework [5,11], the list of *k*-long contiguous substrings (*k*-mers) extracted from given strings is traversed in a* depth-first* search manner with branches corresponding to all possible symbol substitutions from alphabet Σ. Each leaf node at depth *k* corresponds to a particular *k*-mer feature (either exact or inexact instance of the observed exact string features) and will contain a list of matching features from each string. The leaf nodes corresponding to motifs will contain instances from all (or almost all) strings. The complexity of the trie-based traversal algorithm for motif finding is *O*(*k^m^*^+1^|Σ|^m^*nN*). Note that the algorithm essentially explores the neighborhood of all *O*(*nN*) *k*-mers in the input.

Another class of efficient algorithms is based on sorting and enumeration [10]. The PMSP algorithm enumerates all possible neighboring *k*-mers for the first string *s*_1_ and outputs *k*-mers that occur in every string with Hamming distance at most m, similar to the Voting algorithm [8]. The PMSprune algorithm [10] employs a more efficient search strategy to traverse the candidate space and is an improvement, in the expected case, over the PMSP. We note that* explicit enumeration* is employed by all above-mentioned algorithms.

While the exact algorithms focus on retrieving all possible motif patterns, an important issue of estimating significance of the found motif patterns can be addressed with existing techniques as used in, for instance, non-exhaustive algorithms based on stochastic optimization (e.g., MEME [12]).

In contrast to existing exact exhaustive algorithms, we approach the problem of motif finding by performing an efficient search over patterns with wildcards. As a consequence, the proposed method’s complexity becomes independent of the alphabet size.

## Methods

### Combinatorial algorithm for motif search

In this section, we develop an efficient combinatorial algorithm for motif finding with the search complexity independent of the size of the alphabet |Σ|. The algorithm begins by finding a set of candidate motifs, followed by the construction of the intersections of those candidates’ neighborhoods, the sequences that are at most m symbols apart from each candidate pair. In a crucial departure from other approaches, this set is efficiently represented using* stems,* or patterns with wildcards. The number of the stems does not depend on the alphabet size and is a function of the motif length (*k*), the number of mismatches (*m*) and the Hamming distance between *k*-mers. Patterns common to all (or almost all) input strings are then found by pruning the stems that do not satisfy the motif property (i.e., do not occur in all input strings). The main idea of our approach is to construct a candidate set *C* which includes all motifs *M* plus some non-motifs, i.e. *M* ⊆ *C*, and then efficiently select true motifs from the candidate set. Given *C*, the complexity of motif finding is then proportional to its size: the motifs can be extracted from *C* by checking each candidate against the motif property, a task we accomplish using  rounds of counting sort in Algorithm 2. To generate *C*, we collect the sets of stems which characterize the common neighbors of the pairs of *k*-mers (*a, b*) in the input. We call these sets the* stem sets, H*(*a, b*). Finding each *H*(*a*,* b*) is independent of the alphabet size and is accomplished in Algorithm 3. To further reduce the complexity, we construct the stem sets only for potential motif instances *I*, those *k*-mers that are at Hamming distance of at most 2*m* from every input string. We find *I* using  rounds of counting sort (Algorithm 2). We outline our motif search algorithm below:

**Algorithm 1:** Algorithm 1 Motif search algorithm

1. Use multiple rounds of counting sort to iterate over input strings and construct a set of potential motif instances *I*, *k*-mers that are at Hamming distance of at most 2*m* from each string (Algorithm 2).
2. Construct candidate set *C* by building stem sets *H*(*a, b*) for *k*-mer pairs in *I* (Algorithm 3)
3. Prune all stems from *C* that do not satisfy motif property using rounds of counting sort (Algorithm 2, Section ‘Pruning using selection’)
4. Output remaining stems as motifs.

This algorithm uses as its main sub-algorithm (in step 2) a procedure that finds the intersection of *k*-mer neighborhoods for any pair of the *k*-mers *a*, *b*. This intersection finding algorithm is described in Section ‘Motif generation’. We describe selection and pruning steps (steps 1 and 3) in Section ‘Selection algorithm’.

The overall complexity of the algorithm is  where *H* is the maximum size of *H*(*a*, *b*), and *I* is the size of *I*, the number of *k*-mers used to construct the candidate set *C*. The important fact that makes our algorithm efficient in practice is that typically *I* ≪ min(*nN*, |Σ|*^k^*) and *H* ≪ *V*(*k*, *m*), particularly for large alphabets. We demonstrate this in our experimental results and provide an expected-size analysis in Section ‘Selection algorithm’.

### Selection algorithm

A necessary condition for a group of *k*-mers to have a shared, common neighbor (motif) is that the Hamming distance between any pair of patterns cannot exceed 2*m*. We will use this condition to select *k*-mers from input that are potential motif instances and place them in set *I*. A particular *k*-mer *a* in the input is a potential motif instance if it is at the minimum Hamming distance at most 2*m* from each of the input strings. All other *k*-mers that violate the above condition cannot be instances of a motif and can be discarded. To select the valid *k*-mers, we use multiple rounds of count sort by removing iteratively 2*m* out of *k* positions and sorting the resulting set of (*k* − 2*m*)-mers. A *k*-mer is deemed a potential motif instance if it matched at least one *k*-mer from each of the other strings in at least one of the sorting rounds. The purpose of sorting is to group same *k*-mers together. Using a simple linear scan over the sorted list of all input *k*-mers, we can find the set of potential motifs and construct *I*. This algorithm is outlined in Algorithm 2. As we will see in the experiments (Section ‘Results and Discussion’), the selection step

**Algorithm 2:** Algorithm 2 Selection algorithm

**Input:** set of *k*-mers with associated sequence index, distance parameter *d*
**Output:** set of *k*-mers at distance *d* from each input string
1. Pick *d* positions and remove from the *k*-mers symbols at the corresponding positions to obtain a set of (*k* − *d*)-mers.
2. Use counting sort to order (lexicographically) the resulting set of (*k* – *d*)-mers.
3. Scan the sorted list to create the list of all sequences in which *k*-mers appear.
4. Output the *k*-mers that appear in every input string.

significantly reduces the number of *k*-mer instances considered by the algorithm and improves search efficiency. The number of selected *k*-mers, i.e. the size of *I*, is small, especially for large-alphabet inputs. This can be seen from the expected case analysis. For this purpose we assume that sequences are generated from a background process with few motifs implanted in the background-generated sequences. Assuming an iid background model with equiprobable symbols, the expected number of *k*-mers in the input of *N* strings of length n that match each of the *N* strings with up to 2*m* mismatches by chance is 

where *p_k_*_,2_*_m_* is the probability that two randomly selected *k*-mers are at distance of at most 2*m*. For instance, for a set of *N* = 20 protein sequences (sampled from alphabet |Σ| = 20) of length *n* = 600 the expected number of potential motifs of length *k* = 13, *m* = 4 by chance is about 8, with* p*_13,8_ = 2.9 10^−4^. Given *t* implanted motif instances, the average number of *k*-mers that will be selected from *nN* input samples, or the expected size of *I*, is

*E*[*I*] = *t* + *nN*(1 − (1 − *p_k_*_,2_*_m_*)*^t^*) +* E*[*I_B_*].

Since *t* and* p* are typically small, for small *pn*, *E*[*I*] ≪ *nN*, the number of *k*-mers in the input. In the protein example above the expected size of *I* is about 1 + 3 + 8=12 for *t* = 1, which is orders of magnitude smaller than *nN* = 12000, signifying the importance of creating *I* first. This is empirically demonstrated in Section ‘Results and Discussion’.

#### *Pruning using selection*

The sorting approach of Algorithm 2 is also used to select patterns satisfying the motif property from the candidates *C* (Step 3 in main Algorithm 1). The pruning step is based on verifying the motif property (i.e. whether given patterns match all input sequences with up to m mismatches) and can be accomplished using  rounds of counting sort.

### Motif generation

In what follows, we describe an efficient algorithm that finds the set of* stems* that represent the set of *k*-mers shared by a pair of *k*-mers *a* and *b*. This process is used to create set *C* from potential instances *I*, which is subsequently pruned to yield the true motif instances.

The number of *k*-mers in the common neighborhood of any two particular *k*-mers *a* and *b* assumes a fixed set values depending on the Hamming distance *d*(*a*, *b*) between *k*-mers [13], for given values of |Σ|, *k*, and *m*. We want to represent the shared *k*-mers in this intersection using a set of* stems,* patterns with wildcards. However, the number of stems will not depend on the alphabet size |Σ|.

To find all stems shared by *k*-mers *a* and *b*, consider two sets of positions:* mismatch region* in which *a* and *b* disagree and* match region* in which *a* and *b* agree. We consider two cases depending on the number of mismatch positions (i.e. Hamming distance between *a*, *b*). In the first case, the distance *d*(*a*, *b*) is at most *m*, the maximum number of mismatches allowed. In the second case, the distance *d*(*a*, *b*) exceeds *m*. When *d*(*a*, *b*) ≤ *m*, wildcard characters can appear both inside and outside of the mismatch region. When *d*(*a*, *b*) >*m*, wildcard characters can appear only inside the mismatch regions. Consider for example, the case of *d*(*a*, *b*) = 0 and *m* = 1. In this case, the set of stems is the set of patterns with 1 wildcard at each of the possible *k* positions (with the remaining positions as in *a*) plus one stem with 0 wildcards. When *m* = 2, and *d*(*a*, *b*) = 1, the set of stems will include patterns with 0 or 1 wildcard in *k* − *d* positions and 0 or 1 wildcards in the remaining *d* = 1 positions. For example, for the pair (*tgt*, *tgc*) the corresponding patterns with wildcards are *tg*?, *t*??, ?*g*?, *t*?*c*, and ?*gc*, where ? denotes a wildcard.

We outline our algorithm for finding set of stems for the *k*-mer neighborhood intersection in Algorithm 3. The number of stems generated by the algorithm is

The number of stems describing all the explicit *k*-mers shared between *a*, *b* does not depend on the alphabet size. The complexity of the stemming algorithm is proportional to the number of stems generated. The maximum number of stems *H* is  for typical values of *m* <*k*/2. We use Algorithm 3 for every pair of *k*-mers in *I* (step 2) to construct *C* as outlined in the main algorithm.

**Algorithm 3:** Algorithm 3 Stem generation (independent of the alphabet size |Σ|)

**Input:** pair of *k*-mers *a*, *b*
**Output:** set of stems (patterns with wildcards) shared by *a* and *b*
**if** if *d*(*a*, *b*) ≤ *m***then**
Set stem = *a*
Set *i* = 0 … *d* positions in the mismatch region of the stem as in *b*
Place* j*_1_ = 0 … *d* − *i* wildcards inside the mismatch region
Place *j*_2_ = 0 … *m* − max(*d* − *i*, *j*_1_ + *i*) wildcards outside the mismatch region
**end if**
**if***d*(*a*, *b*) >*m***then**
Set stem = *a*
Fix *i* = *d* − *m* … *m* positions in the mismatch region of the current stem as in *b*
Place *j* = 0 … *m* − *i* wild-cards in the remaining *d* − *i* positions in the mismatch region
**end if**
Output resulting stems (patterns with wildcards)

### Algorithm analysis

The complexity of the selection step 1 for constructing *I* is  and does not depend on the alphabet size |Σ|. Steps 2 and and 3 have the complexity  and again do not depend on |Σ|. As a consequence, the three-step procedure gives us an efficient, alphabet-independent motif search algorithm that outputs all motifs embedded in the input *S*. Our experiments will next demonstrate that this allows efficient exploitation of sparsity of typical solutions—we explore only a small portion of the motif space by focusing (using Algorithm 2) only on the support samples that are potential instances of the motifs. This results in significant reductions in running times, especially for large-alphabet inputs, i.e. the cases difficult for the current exact motif finding algorithms.

### Extensions

Our proposed framework can be used to reduce search complexity for other exact search-based motif finding algorithms. Existing exhaustive algorithms typically (e.g. [5,8,10]) use the entire input (i.e. all the *k*-mers in the input) and find motif by essentially exploring neighborhoods of every *k*-mer in the input. Their search complexity can be improved by using a* reduced* set of *k*-mers instead of all input samples. This reduced set of *k*-mers can be obtained using our linear time selection algorithm (Algorithm 2, Section ‘Selection algorithm’). Using reduced set of *k*-mers, the actual search complexity after the selection step becomes sublinear in the input size (since the number of selected *k*-mers *I* = |*I*| is much smaller than input length *O*(*nN*)). For instance, the search complexity of the trie-based algorithms (e.g., [5]) can be reduced to  instead of *O*(*knNV*(*k*, *m*)), where *V*(*k*, *m*) is *O*(*k^m^*|Σ|*^m^*). This will lead to a more efficient search especially for large-alphabet since a possibly large input (*O*(*knN*)) is replaced with a smaller set *I* of *k*-mers that match with up to 2*m* mismatches every string in the input.

## Results and discussion

We evaluate our algorithms on synthetic benchmark motif finding tasks and real data sets. We first test our algorithms on the planted motif problem commonly used as a benchmark for evaluation of the motif finding algorithms [2,5,10]. We then illustrate our method on several DNA and protein sequence data sets.

### Planted motif problem

A planted motif problem [2] is the task of finding motifs and their instances in a set of sequences with variants of the consensus string (motif) implanted with up to *m* mismatches in every string. This task represents a well-defined subtle motif discovery problem. Instances of this problem with large number of mutations *m* are known to be challenging for most of the motif finding algorithms.

We follow the standard setting used in previous studies [2,5,10] and generate *N* = 20 random strings of length *n* = 600 using iid, uniformly distributed symbols from an alphabet of size |Σ|. We then embed a copy (with up to *m* substitutions at random positions) of a motif at a random location in every string. The task is then to identify motifs hidden in the input.

**Figure 2:**
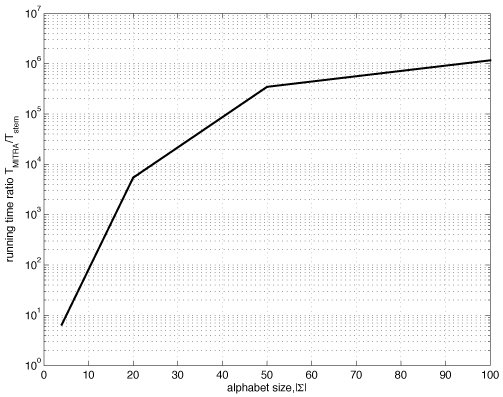
Running time ratio (*T_MITRA_*/*T_stem_*) as a function of the alphabet size (planted motif problem, *k* = 13, *m* = 4), logarithmic scale

In Table 2, we compare the running time of our algorithms with state-of-the-art motif finding algorithms on several challenging instances of the planted motif problem. We give the running time comparison for large-alphabet (|Σ| = 20 − 100) instances in Table 3. As we can see from the results in Table 2 and Table 3, our algorithms show significant reduction in running times compared to state-of-the-art methods, especially for large-|Σ| inputs (Table 3). For large alphabets and large *k*,*m* trie traversal takes substantial amount of time and results in these cases are not reported. In Figure 2, we show the running time ratio (logarithmic scale) between the mismatch trie traversal (MITRA) algorithm and our algorithm as a function of the alphabet set size. The running time is measured on (13,4) instances of the planted motif problem. For relatively small alphabet of size 20 our algorithm is about 10^4^ times faster than the mismatch trie. The difference in running time increases with the size of the alphabet. Large alphabets can, for instance, arise when encoding the 3D protein structure, a necessity in cases when sequences share little similarity at primary level.

**Table 2:** Running time comparison on the challenging instances of the planted motif problem (DNA, |Σ| = 4, *N* = 20 sequences of length *n* = 600). Problem instances are denoted by (*k,m,* |Σ|), where* k* is the length of the motif implanted with *m* mismatches.

Motif problem instances (*k*, *m*, |Σ|)						
**Algorithm**	**(9,2,4)**	**(11,3,4)**	**(13,4,4)**	**(15,5,4)**	**(17,6,4)**	**(19,7,4)**

Stemming	0.95	**8.8**	**31**	**187**	**1462**	**8397**
MITRA [5]	**0.89**	17.9	203	1835	4012	n/a
PMSPrune [10]	0.99	10.4	103	858	7743	81010
RISOTTO [6]	1.64	24.6	291	2974	29792	n/a

**Table 3:** Running time, in seconds, on large-|Σ| inputs. (*k*, *m*) instances denote implanted motifs of length *k* with up to *m* substitutions.

|Σ|	(9,2)		(11,3)		(13,4)		(15,5)	
	**MITRA**	**Stemming**	**MITRA**	**Stemming**	**MITRA**	**Stemming**	**MITRA **	**Stemming**

20	8.39	**0.637**	1032.17	**1.07**	28905	**5.247**	n/a	**12.31**
50	89.82	**0.633**	12295.73	**0.963**	685015	**2.244**	n/a	**11.92**
100	265.94	**0.645**	n/a	**0.967**	> 1 month	**2.227**	n/a	**11.86**

**Figure 3:**
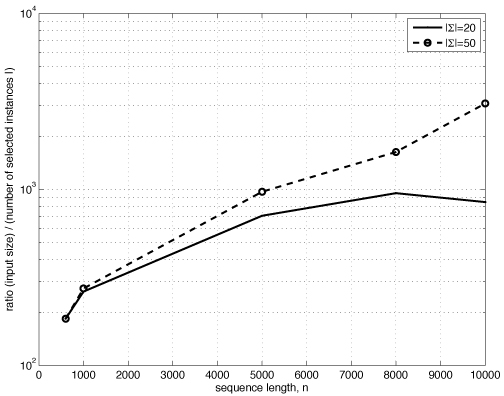
Ratio between input size (*nN*) and the number of selected samples (*I* = |*I*|) as a function of the input length and alphabet size (planted motif problem, *k* = 13, *m* = 4). Note logarithmic scale.

Figure 3 shows efficiency of the selection (step 1 in the algorithm) as a ratio between the input size and the number of the selected samples (*k*-mers) |*I*|. We observe that across different input sizes selection reduces the number of samples by a factor of about 10^3^. The observed number of selected samples *I* = |*I*| agrees with the theoretical estimates (Section ‘Selection algorithm’) (e.g., for |Σ| = 50, n=5000, N=20, we expect about 52 *k*-mers to be selected, and the observed size of *I* is 103 *k*-mers).

### Identifying TF binding sites

We use several data sets with experimentally confirmed TF binding sites: CRP, FNR, and LexA. The CRP data set contains 18 DNA sequences of length 105 with one or two CRP-binding sites [2,14]. The FNR and LexA data sets are obtained from RegulonDB [15] database and contain 30 and 91 sequences known to have sites of length 14 and 20 bases. The task is to identify the sequence motif corresponding to the binding sites and the positions of sites within sequences.

For CRP, we use relatively long *k*-mers of length *k* = 18, with a large number of allowed mismatches (*m* = 7) from a given set of 18 DNA sequences (|Σ| = 4). For FNR and LexA data sets, we set motif length to *k* = 14 and *k* = 16 bases, with the maximum number of mismatches set to *m* = 4 and *m* = 6, respectively.

**Figure 4:**
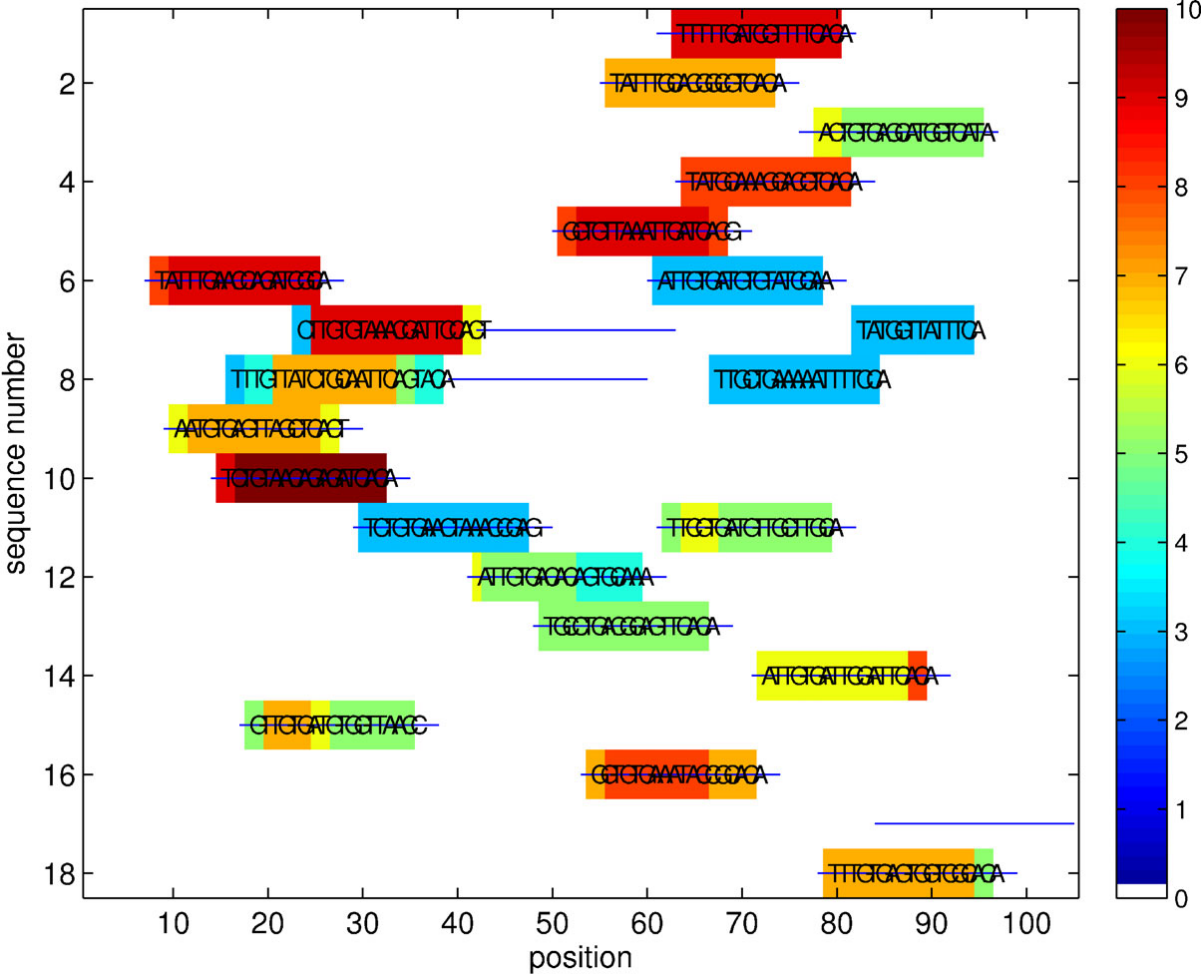
Recognition of CRP binding sites (*k* = 18, *m* = 7, |Σ| = 4)

Figure 4 illustrates motifs found by the algorithm on the CRP data set. In the figure, colors indicate the importance of positions as measured by the number of hits between the found motif patterns and the sequences, with blue horizontal lines denoting true (confirmed) locations of the binding sites. The set of discovered locations agrees with the set of experimentally confirmed primary positions. The discovered motif patterns correspond to instances of the reference consensus motif TGTGAnnnnnnTCACA [14,16]. Because of large *k* and *m* we observe running time improvements similar to the benchmark planted motif problems: our algorithm takes about 6 minutes, while the mismatch trie traversal requires about 12 times as long (4489 seconds). Allowing a large number of mismatches (*m* = 7) in this case is critical for the motif prediction performance, because fewer mismatches do not lead to successful identification of the binding sites.

For FNR and LexA motifs, our algorithm correctly finds consensus patterns TTGATnnnnATCAA and CTGTnnnnnnnnnCAG, in line with the validated transcription factor binding sites, with the performance coefficients [2] of 83.69 and 90.38.

### Protein motif finding

We also apply our algorithm to finding subtle sequence motifs on several protein sequence datasets, a challenging task due to the increased alphabet size (|Σ| = 20) coupled with large *k* and *m*.

**Figure 5:**
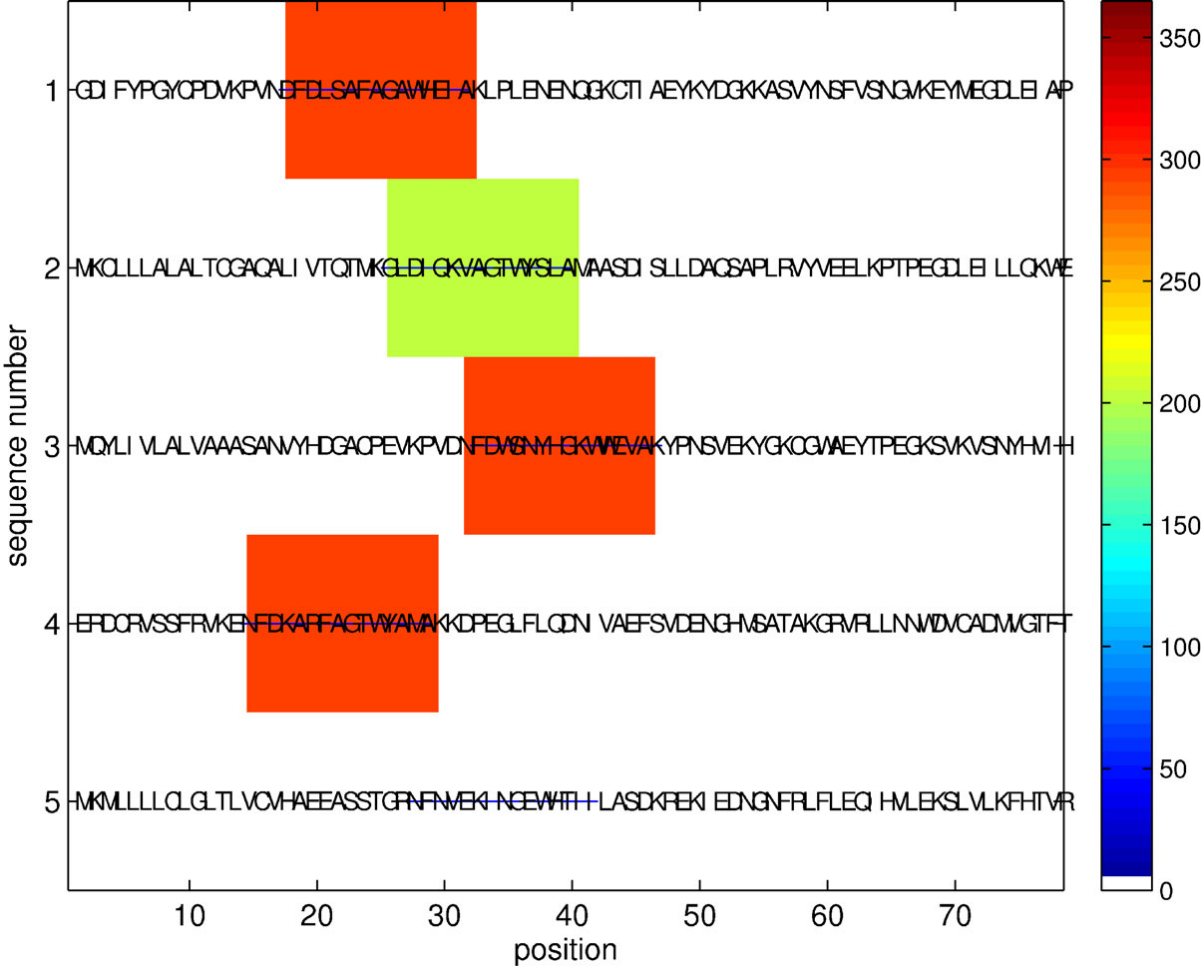
Lipocalin motifs (*k* = 15, *m* = 7, |Σ| = 20)

**Lipocalin motifs.** We first consider motifs in* lipocalins* which are topologically similar but have very diverse primary sequences. Using *k*-mer of length *k* = 15 with *m* = 7 mismatches, we identify motifs containing 15 residues with the instance majority FD[IKLW]S[AKNR]FAGTWYE[ILMV]AK (Figure 5), which agrees with the known reference motif [17]. Our algorithm takes about 5 minutes to complete this task, while the mismatch trie algorithm takes more than a day. As in the case of the DNA, a large number of mismatches is critical for finding motifs, while smaller values of *k*, *m* do not result in motif identification. 

**Zinc metallopeptidase motif.** In this experiment, 10 relatively long (average length is 800) human zinc metallopeptidase sequences used to test motif finding. Identification of subtle motifs in this case is made even more challenging by the length of the sequences. We use 11 residues long *k*-mer with *m* = 5 mismatches and find sequence motifs with the instance majority VAAHELGHS[GL]G in 9 out of 10 sequences that correspond to previously confirmed locations. We note the large number of mismatches (*m* = 5) was critical to motif identification.

**Figure 6:**
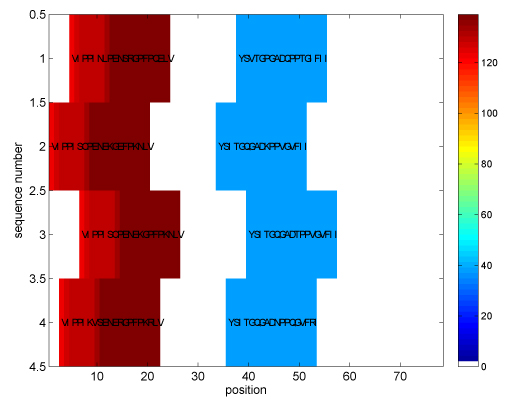
The sequence patterns for supersecondary structure motifs (Cadherin superfamily, *k* = 20, *m* = 4, |Σ| = 20)

**Super-secondary structure sequence motifs.** We consider now two data sets of protein sequences with interesting 3D sandwich structure studied previously by biologists, for which existence of corresponding sequence motifs has been postulated [18]. Using Cadherin and Immunoglobin superfamilies as an example, our algorithm finds sequence patterns that correspond to the supersecondary structure (SSS) motifs [18,19], i.e. arrangements of the secondary structure units (loops, strands). In particular, in Cadherin superfamily we find long motifs of length 20 (using *m* = 4 mismatches) corresponding to the secondary structure units* strand 1 - loop - strand 2* (VIPPISCPENE[KR]GPFPKNLV) and* strand 3 - loop - strand 4* (YSITGQGAD[KNQT]PPVGVFII) (3D SSS motif [19]). Figure 6 shows identified motif positions within the sequences. Our algorithm finds 36 potential motif instances (out of 330 samples) after the selection (step 1) and takes about 47 seconds (compared to about 600 seconds using the trie traversal). In Immunoglobin superfamily (C1 set domains), we find a sequence motif of length 19 SSVTLGCLVKGYFPEPVTV which corresponds to* strand 2-loop-strand 3* secondary structure units (2E SSS motif).

## Conclusions

We presented a new deterministic and exhaustive algorithm for finding motifs, the common patterns in sequences. Our algorithm reduces computational complexity of the current motif finding algorithms and demonstrate strong running time improvements over existing exact algorithms, especially in large-alphabet sequences (e.g., proteins), as we showed on several motif discovery problems in both DNA and protein sequences. The proposed algorithms could be applied to other cases and challenging problems in sequence analysis and mining potentially characterized by large alphabets, such as text mining.

## Competing interests

The authors declare that they have no competing interests.

## Authors contributions

All authors contributed equally to this work.
